# Olfactory and gustatory outcomes following endoscopic transsphenoidal hypophysectomy

**DOI:** 10.1186/s43163-023-00426-y

**Published:** 2023-04-17

**Authors:** Indumathi Ainer, Salina Husain, Aneeza Khairiyah Wan Hamizan, Farah Dayana Zahedi, Jegan Thanabalan

**Affiliations:** 1 Department of Otorhinolaryngology, UKM Medical Centre, Kuala Lumpur, Jalan Yaacob Latiff, Bandar Tun Razak, Cheras, 56000 Malaysia; 2 Department of Surgery, Neurosurgical Unit, Faculty of Medicine, Universiti Kebangsaan Malaysia, Kuala Lumpur, Cheras, 56000 Malaysia

**Keywords:** Olfaction, Taste, Pituitary Adenoma, Hypophysectomy, Endoscopy

## Abstract

**Objective:**

The aim of this study was to evaluate olfactory, gustatory, and quality-of-life outcomes in patients who underwent endonasal transsphenoidal hypophysectomy.

**Methods:**

In this prospective study, the patients were assessed subjectively using the Malay version of sQOD-NS (short questionnaire of olfactory disorders in a negative statement) and objectively using the culturally adapted Sniffin’ Sticks smell test and taste test preoperatively and 3 months postoperatively. The Sniffin’ Sticks smell test consists of odor identification, odor discrimination, and odor threshold tests. The taste test consists of different sweet, salt, sour, and bitter concentrations.

**Results:**

Twenty patients were enrolled in the study. The study comprises 45% female and 55% male. On average patients’ ages were 49.5 years. In this study we found a significantly reduced in odor identification score (*p* = 0.049) post-surgery; however, there was no statistically significant difference in odor threshold, odor discrimination, and taste. The olfactory quality of life outcome based on the Malay version of sQOD-NS (*p* = 0.001) was significantly reduced after surgery. There was no significant difference in the Sniffin’ Sticks smell test (*p* < 0.178) and taste test (*p* < 0.425) pre-surgery and post-surgery. The tumor’s location, either sellar or suprasellar, did not influence the smell outcome of patients postoperatively (*p* = 0.056).

**Conclusion:**

The study showed that the endoscopic transsphenoidal technique for pituitary surgery does not pose permanent olfactory disability.

## Background

Olfaction is an important sense used in daily life. Olfaction plays a big role in tasting food and avoiding harmful substances like fire and eating spoiled food [1]. Therefore, olfactory dysfunction reduces the quality of life and can also be life-threatening [2].

Endonasal transsphenoidal hypophyseal surgery can alter natural physiologic conditions consequently increasing postoperative sinonasal morbidity resulting in a negative impact on a patient's daily life [3].

There have been only a few studies that have attempted to evaluate olfactory function after endoscopic transsphenoidal surgery, and the results have been conflicting with some studies showing profound impairment and others showing no loss or only minimal loss of function. Moreover, only a few studies have addressed quality of life (QOL) with regard to smell and taste in patients treated surgically [4].

Little has been published to date on the short-term QOL outcomes, specifically sinonasal measures, after endoscopic pituitary surgery. Lee et al. [5] concluded that they were marked improvement in emotional well-being after surgery. Jemma Cho et al. concluded that nasal function, sinonasal treatments, and medication use on disease-specific QOL surveys indicate the endoscopic approach had lesser morbidity [6].

Suberman et al. stated that endoscopic, endonasal pituitary surgery with good postoperative management results in minimal or no long-term sinonasal QOL disturbances. In relation to the Questionnaire of Olfactory Disorders-Negative Statements (QOD-NS), many studies have been done on novel coronavirus-19 (COVID-19) where patients frequently complained of ageusia and anosmia with relation to psychosocial aspects [7]. Fan Yaun et al. in 2022 stated in chronic rhinosinusitis (CRS) patients, the olfactory-specific QOL was significantly correlated with TDI scores. sQOD-NS in patients with CRS had high validity and a strong association with objective olfactory metrics and olfactory cleft assessment [8].

Rong-San et al. studied the gustatory function and its effect on functional endoscopic sinus surgery (FESS). This study concluded that the whole mouth suprathreshold taste test (WMTT) sweet and bitter scores were significantly lower after FESS in CRS with polyposis while the taste quad test (TQT) sweet score was significantly higher in patients CRS without nasal polyposis [9].

The aim of this study is to evaluate to smell and taste outcome post transnasal endoscopic transsphenoidal hypophysectomy. Smell outcome was assessed both objectively using Sniffin’s Stick test and subjectively using the Malay version of the sQOD-NS questionnaire. In this study, we have also incorporated taste outcomes as a part of research to determine any disturbances in taste postoperatively.

## Methods

This study was a prospective study conducted at a single center. Approval was obtained from the Ethics Committee of Universiti Kebangsaan Malaysia Medical Centre (UKMMC) and funding from grant Fundamental Grant of the National University of Malaysia (UKM PPI /111/8/JEP 2021/126). This study was performed in accordance with the Declaration of Helsinki for research on human subjects. Participants were recruited from UKM Medical Centre who were admitted for surgery and fulfilled all the inclusion and exclusion criteria. A total of 20 subjects were recruited for this study. Patients with pre-existing olfactory or gustatory dysfunction, history of major head trauma, usage of oral and topical steroids up to 6 weeks before the operation, acute bacterial or viral infection and sinusitis, with previous nasal surgery and postoperative cerebrospinal fluid leak were excluded from the study. Informed consent was taken from the participants. Participants were grouped before and after surgery. Demographic data were collected for each participant.

The Malay version of sQOD-NS is a validated basic questionnaire used to assess the olfactory-specific quality of life. This is a patient-reported outcome questionnaire including social, eating, annoyance, and anxiety questions. Question 1—change in smell affects social outings, 2—impact on daily social activities, 3—problems with my sense of smell make me more irritable, 4 and 5—smell associated with eating habits, 6 and 7—for anxiety related to change in smell. Items are rated on a scale of 0–3, with higher scores reflecting a better olfactory-specific quality of life (QOL). The total score ranges from 0 (severe impact on QoL) to 21 (no impact on QoL). The item and total scores of sQOD-NS significantly differ between patients with presumed anosmia at the time of the assessment, and those with presumed hyposmia or without olfactory dysfunction [10].

Patient’s olfactory ability was assessed using the identification, discrimination and threshold Sniffin’ Stick tests. The identification test which was used consist of a culturally adapted Malaysia version of the Sniffin’ Sticks test. The technique of using the pen is to place the tip approximately 2 cm in front of both nostrils of participants for around 3 s. There are three distractors and one correct odorant (four in total) which are presented in the pictorial form in front of the participant. Participants must identify one odorant; if he/she is unable to identify the odor, they are required to re-smell the stick and force choose one answer. The identification process will continue until all 16 pens are completed. The time interval between 2 odorant pens will be 20–30 s.

For discrimination and threshold test, participants were required to be blindfolded using a sleep mask. A set of sixteen triplet pens are labeled green, blue, and red cap numbers from 1 to 16. The tests demanded participant concentration. In the discrimination test, triplet pens, two of the pens contained the same odor, target pens to be discriminated and it is green caps. Participants were asked to identify the target pen. Using a verbal answer by stating “first,” “second,” or “third” after taking a sniff. The order of the pen is alternated to reduce bias.

For the threshold test, sixteen sets of triplet pen concepts were used. The target pen is a red cap instilled with* n*-butanol, and two distractor pens were blank. The target pens have different concentrations; set number 16 has the lowest concentration and set number 1 has the highest concentration. The participants were familiarized using *n*-butanol odor using a number 1 pen. Participants were asked to identify which is the pen with *n*-butanol. The order starts from the lowest concentration, and a staircase paradigm was utilized until to correct answers were obtained; this is marked as a first turning point. After the first point was found, the next higher dilution step was offered and not correctly identified target pen was considered as a second turning point. The third turning point was identified when participants correctly identifies twice in a row with triplets with higher concentration. The same steps are repeated until 7 turning points are achieved. The threshold is the mean of the last 4 turning points. The TDI score is a sum of the score for the olfactory ability of threshold, discrimination, and identification.

The Burghart taste strips consist 19 × 50 strips and are a validated examination procedure to investigate the taste ability. Taste strips are touched on the tongue and the patient may close the mouth and move the tongue. If there is interest in the taste sensitivity of certain tongue areas, the mouth stays open and the strip will only be in contact with this area until the patient can give an answer. The complete taste strip set consists of 16 containers with 4 concentrations of sweet, sour, salty, and bitter each and 3 containers with blanks. The results of this test reflect a measure of the ability to identify 4 basic tastes. The test–retest reliability compares well with other taste tests.

The gustatory function was assessed using the validated “Taste Strips” test (Burghart Medical Technology, Wedel, Germany). The test consists of 16 filter paper taste strips that were applied in a randomized sequence (4 concentrations of each of the 4 basic taste qualities: *sweet*: 0.4, 0.2, 0.1, and 0.05 g/mL sucrose; *sour*: 0.3, 0.165, 0.09, and 0.05 g/mL citric acid; *salty*: 0.25, 0.1, 0.04, and 0.016 g/mL sodium chloride; *bitter*: 0.006, 0.0024, 0.0009, and 0.0004 g/mL quinine hydrochloride). The test was started with the lowest concentrations, in order to minimize adaptation and habituation processes. A total of 16 scores will be recorded and randomly tested.

### Statistical analysis

The data were analyzed with SPSS version 26.0 SPSS 26.0 (IBM, Armonk, New York). The data were descriptively analyzed to get the median of sQOD-NS, TDI, and taste scores. Data were then further explored by examining for statistical significance of preoperative and postoperative results.

The frequency testing was used to compare TDI scores between gender and age. A Wilcoxon signed-rank test was used to compare preoperative and postoperative results which include sQOD-NS, TDI, and taste scores as the data is not normally distributed. The correlation between sQOD-NS questionnaire score and TDI scores using Pearson’s correlation coefficient test. A comparison between sellar and suprasellar lesions with TDI scores was done using the chi-square test. A *p* value of less than 0.05 is regarded as statistically significant.

### Surgical technique

Pituitary tumor surgery through a combined approach carried out by a neurosurgeon and otorhinolaryngologist via an endoscopic transsphenoidal approach. A rhinologist started the surgery. The patient was put in neutral position as best exposure to the skull base. Nasal decongestion was done using packing with ribbon gauze impregnated with tranexamic acid 500 mg /5 ml and co-phenylcaine 1 ml inserted into both nostrils using headlight and thudicum speculum and Tilley’s forceps. The middle turbinate cautiously lateralized and sphenoid ostium was identified. The nasoseptal flap was elevated and kept at the nasopharynx. The sphenoid ostium was enlarged in the middle and the posterior. During this procedure, care was taken not to damage the posterior septal branch of the sphenopalatine artery. The sphenoid sinus wall was removed and a posterior septectomy was performed. The thickened bone was trimmed using a drill. The intersinus septum removed with care and it may be laterally placed and adhered to the carotid artery. The mucosa of the sphenoid wall cleared. Then, sella was approached using a drill. For the suprasellar tumor, bilateral posterior ethmoidectomy was performed and the planum of the sphenoid sinus was exposed. The procedure was then passed to a neurosurgeon. Tumor clearance was done and a histological sample was taken. Hemostasis performed. Duragen and duraseal are used to close the defect. If the nasoseptal flap is raised, it is placed overlying the defect to close it. Some cases used abdominal fat plugging.

## Results

Indications for surgery include a non-functioning pituitary macroadenoma in 18 patients (85%), 2 craniopharyngiomas (10%), and 1 Rathke’s cleft cyst (5%). Three patients had CSF leaks intraoperatively. An immediate repair was performed. No recurrence of leak postoperatively. There was no postoperative synechiae, or septal hematoma observed.

A total of 20 patients were included in this study. The mean age was 49.2 ± 14.2 (age range 21- 72 years). In this study, female showed a 45% [9] and male gender is 55% [11] from the study.

### sQOD-NS questionnaire

There was a statistically significant difference between preoperative and 3 months postoperatively (*p* = 0.001). The mean scores were 18.05 ± 2.26 and post 16.60 ± 1.53. The study concurred olfactory quality of life outcomes of patients were affected after surgery when tested at 3 months (refer to Table 1: statistical comparison using preoperative and postoperative non-parametric test using Wilcoxon signed-rank test for sQOD-NS questionnaire).

**Table 1:** Statistical comparison using preoperative and postoperative non-parametric test using Wilcoxon signed-rank test for sQOD-NS questionnaire

	Pre-op QOD-NS	Post-op QOD-NS	*p* value
N	20	20	
Mean	18.05	16.60	0.001
Std. deviation	2.259	1.536	

### Threshold discrimination identification (TDI) score

A Wilcoxon Signed Rank Test for non-parametric analysis was used to compare pre and postoperative odor identification, odor discrimination, odor threshold, and total TDI scores. All tests were 2-tailed and a *p-*value of less than 0.05 was considered as significant study. The classification of TDI scores defined functional anosmia as a TDI score ≤ 16.5, normosmia as a TDI score > 30.5, and hyposmia as a score between these two values.

The mean values of the result from the Sniffin’ Sticks smell test performed pre and postoperatively at 3 months were compared for patients who underwent endoscopic transsphenoidal hypophysectomy for pituitary adenoma.

All patients were tested to be normosmic preoperatively with a baseline Sniffin’ Stick smell test carried out and included in the study. There were no patients excluded from the study.

A statistical difference (*p* = 0.049) was found in the preoperative and postoperative odor identification test values, although there was no significant difference in odor threshold (*p* = 0.593) and odor discrimination (*p* = 0.257). There was no significant difference between the pre and postoperative TDI scores of olfactory functions *(p* = 0.178). The overall sense of smell of the patients who were preoperatively normosmic was not affected postoperatively (refer to Table 2: statistical comparison using preoperative and postoperative non-parametric test using Wilcoxon signed-rank test).

**Table 2:** Statistical comparison using preoperative and postoperative non-parametric test using Wilcoxon signed-rank test

Sniffin’ Stick Test	Preoperative mean value (*N* = 20)	Postoperative mean value (*n* = 20)	*p* value
Odor identification	12.15 ± 1.66	11.50 ± 1.63	0.049
Odor discrimination	12.95 ± 1.47	12.80 ± 1.36	0.257
Odor threshold	10.39 ± 1.72	10.56 ± 1.32	0.593
TDI score	35.4 ± 3.08	34.8 ± 3.01	0.178

### Taste test

The mean for preoperative results was 12.70 ± 1.261, and postoperative at 3 months was 12.50 ± 1.357. There was no statistical difference between both tests (*p* = 0.425) (refer to Table 3: statistical comparison using preoperative and postoperative non-parametric test using Wilcoxon signed-rank test for taste).

**Table 3:** Statistical comparison using preoperative and postoperative non-parametric test using Wilcoxon signed-rank test for taste

	Preoperative taste test	Postoperative taste test	*p* value
*N*	20	20	
Mean and SD	12.70 ± 1.261	12.50 ± 1.357	0.425
Median	13.00	12.50	

There is a negative correlation between smell score and sQOD-NS score when a test was performed (refer to Table 4: The correlation between sQOD-NS questionnaire score and TDI score using Pearson’s correlation coefficient test (T: threshold, D: discrimination, I: identification).

**Table 4:** The correlation between the sQOD-NS questionnaire score and TDI score using Pearson’s correlation coefficient test (*T*, threshold; *D*, discrimination; *I*, identification)

Pearson correlation	TDI score	T score	D score	I score
sQOD-NS score	0.134	0.351	0.568	0.178

### Sellar and suprasellar extension with smell outcome

Differences in outcome variables by tumor location were tested using Fisher’s exact test (Table 5: postoperative smell outcome categorized co-relation with tumor location using Fisher’s exact test). All patients who underwent endoscopic transsphenoidal approaches showed (*p* = 0.056) no significant difference of smell outcome when compared with tumor location. Two patients showed a decrease in smell according to TDI scoring.

**Table 5:** Postoperative smell outcome categorized co-relation with tumor location using Fisher Exact test

Post TDI	Sellar	Suprasellar	*P* value
Anosmia	0	0	
Hyposmia	0	2	
Normosmia	8	10	
Total	8	12	0.056

## Discussion

The smell and taste sensation is the least researched compared with other senses, however, the role it plays in our daily life is enormous. The quality of life will be affected when the olfaction is disturbed [12].

There are the previous study has provided information on olfactory outcomes after endoscopic transsphenoidal surgery using the Sniffin’ Sticks smell test [13]. However, to our knowledge, there was no study has been carried out for the evaluation of odor, taste, and quality of life questionnaire using sQOD-NS in a single study.

The pituitary hypophysectomy over the years was performed transcranially and later microscopically. Over the last two decades, the endoscopic method has replaced microscopes. [14].

The Sniffin’s Sticks smell test is a familiar test in Europe. This test was introduced and developed by Hummel et al. in 1997 [15]. It contains 1 test at the threshold and 2 tests above the threshold (identification and discrimination). This kit is user-friendly and can be reused. In Malaysia, the test has been modified according to cultural adaptation [16]. This test was also tested among the Malaysian population with different age groups, and normative values were obtained [17]; hence, its suitability is to be applied to the local population.

The odorants utilized in the Sniffin’s Sticks are those that are culturally adapted and validated in Lum et al. study. This test was performed preoperatively on twenty patients who consented to participate as a study population. In the third month after the endoscopic transsphenoidal surgery, the test was repeated. A significant difference in postoperative identification test score was revealed; however, there was no statistically significant difference in odor threshold and odor discrimination. The result showed that the ability to identify odorants was significantly reduced in patients post-transsphenoidal surgery. However, their ability to discriminate and perceive odorants were not affected. In regards to the identification test, patients are familiar with the odorant given as it was an odorant that may be previously experienced in regular households. In comparison to identification, threshold, and discrimination test, the threshold and discrimination test patients were blindfolded and unable to use a visual cue to get an answer while in the identification test, they were given a card consisting of the answer in visual card form and they were instructed to choose the correct odorant. This is the factor that leads to a significant difference in odor identification tests as a patient were using visual sense and from previous experience with odorant [18]. There was no significant difference between the TDI scores measured. 18 patients were normosmic pre- and postoperatively. 2 patients had hyposmia post-operatively. These 2 patients had pituitary adenoma with suprasellar extension. It may be due to the removal of mucosa containing olfactory epithelium in the posterior olfactory cleft in order to expose the planum during surgery.

Hart et al. in 2010 studied the olfactory outcome post-endoscopic surgery. The University of Pennsylvania Smell Identification Test UPSIT was performed preoperatively, at 1 month, and at 3 months. The mean score (total 40) was 31.8, 30.5, and 32.6 respectively. The result was found to be statistically significant at 1 month and found that olfaction ability improved at 3-month intervals. The study concluded that the damages to olfactory mucosa were transient in nature [18].

In a study conducted by Harvey J et al., [19] a Smell Identification Test (SIT40) was utilized to assess the olfaction ability. There was no difference in SIT40 scores between those who subjectively rated their smell lower or higher at 6 months.

Irene et al. [20] studied olfactory dysfunction between endoscopic and microscopic pituitary surgery preoperatively using the University of Pennsylvania Smell Identification Test (Sensonics Inc., Haddon Heights, NJ). Olfactory function was re-assessed at 6 months and showed a better outcome in the endoscopic group.

Yangseop Noh et al., [21] studied the preoperative and 3 months postoperative using Cross-Cultural Smell Identification Test (CC-SIT) resulted in no difference in smell outcome.

Kikuchi et al. in 2020 [22] studied 26 patients who underwent surgery for pituitary tumor endoscopically. The olfactory outcome was assessed by T&T olfactometer before and 6 months after surgery. The mean recognition threshold values of T&T olfactometer significantly improved after surgery (*P* = 0.01).

Pu Li et al. in 2020, conducted a retrospective cohort study on 232 patients who underwent endoscopic endonasal transsphenoidal surgery (EETS) for pituitary adenoma. Two methods of superior turbinate (ST) management were used; partial resection of ST and lateralization of the ST. A threshold test and 12-item identification test from the Sniffin’ Stick test was traced from preoperative and 6 months postoperative, showed the partial resection of the ST group, and showed a significantly lower score in the threshold test. However, no significant difference in the identification test (*p* = 0.325) in both groups. In conclusion, the overall smell outcome does not change in the partial resection of ST [23].

Kim et al. in 2014, conducted at a prospective study of 226 patients who underwent endoscopic hypophysectomy binostril approach. They were assessed preoperatively and 6 months postoperatively for smell outcome. These sample populations were classified into 2 groups according to the surgical approach. In the first group, a conventional nasoseptal flap was created along the sagittal plane of the septum and the inferior border of the sphenoid ostium. In the second group, a curvilinear incision was made along the inferior border of the sphenoid ostium, anteriorly to the level of the middle turbinate along the septum far away from the olfactory mucosa. The result showed olfactory score was less severe in the second group. This shows that type of nasoseptal flap may influence the olfactory outcome.

Many studies have shown a minimal or insignificant change in outcome in olfaction after the endoscopic hypophyseal operation [24]. In this study, the binostril approach was advocated. The superior border of posterior septectomy was at the level of the sphenoid ostium except for the removal of the suprasellar tumor whereby the procedure was extended above the sphenoid ostium. The mucosa containing olfactory receptors in the posterior part of the olfactory cleft was removed just to provide the access to the superior part of the sphenoid rostrum and planum. None of sellar tumors had hyposmia/anosmia postoperatively; however, 2 out 12 patients with suprasellar tumors had hyposmia. Even though the olfactory test pre- and post-surgery was not significant, it is important to preserve olfactory epithelium in the posterior olfactory cleft during suprasellar tumor removal.

The sinonasal outcome using the sQOD-NS questionnaire was comparable in this study. This result indicated that transsphenoidal surgery did affect the quality of life in relation to smell dysfunction. There was no study conducted using the sQOD-NS questionnaire on patients post transsphenoidal surgery. Previously it was used for COVID-19 patients and recently on sinusitis patients.

The consequence of endoscopic pituitary surgery is debatable. Sowerby et al., have reported no disturbances in olfaction after endoscopic pituitary surgery [25] using a 22-item Sino-Nasal Outcome Test (SNOT-22), and objectively via the Lund-Kennedy Endoscopic Scoring system (LKES), whereas other researchers have concluded significant changes in olfactory function after endoscopic transsphenoidal surgery [26]. What QoL questionnaire used?

In a recent study by Charlie et al. [27], the General Nasal Patient Inventory (GNPI) was prospectively administered to 136 patients. The GNPI is a sensitive tool used in evaluating pre- and postoperative nasal symptoms after nasal surgery. There was no significant score difference in endoscopic transsphenoidal patients after 1–2 weeks postoperatively compared with preoperatively [28]. However, the extent of tumor surgery was not mentioned.

Novák et al. [29] recruited 65 patients, 33 male and 32 female who underwent endoscopic endonasal surgery due to sellar expansion. A sinonasal quality of life was evaluated using the Sinonasal Outcome Test-22 (SNOT-22) questionnaire used to measure the sinonasal outcome. It was concluded there was no significant difference in QOL between scores before and 6 months after surgery [27].

Nanki Hura et al. [30] conducted a study with 46 patients with nonfunctioning pituitary adenoma(*n* = 28). ASK nasal-12 score was used a tool to demonstrate before and after surgery sinonasal outcome. There was a worsening of subjective smell (mean = 0.62) and taste function (mean = 0.42) postoperatively, which persisted at approximately 3 months postoperatively (*P* = 0.0059) [29].

The taste test using the taste test strip showed no significant difference pre- and post-surgery. Patients were able to identify sweet tastants better than other tastants.

The attained values refer entirely to the anterior two-thirds of the tongue (innervated by chorda tympani) [30]. In the posterior two-third of the tongue, a gag reflex can be elicited and cause discomfort to patients. The taste buds at the anterior two-thirds of the tongue have the highest density of taste buds usually implicating gustatory dysfunction [31].

In a study by Bedrosian et al. [32], an Anterior Skull Base Questionnaire (ASBQ), a validated QOL instrument used to measure postoperative taste, smell, appetite, nasal secretions, and vision before anterior skull base surgery and postoperative 1 year. Both taste (*p* = 0.03) and smell (*p* ≤ 0.001) were significantly decreased by 6 weeks postoperatively. At 12 months both taste and smell scores returned to the normal range.

Our study showed the taste outcome was generally undisturbed at 3 months after surgery in relation to the total score. There was no specific Taste test strip test performed in patients who underwent pituitary surgery and published as a study before.

## Conclusion

Our study found that the transsphenoidal surgery did not affect the overall senses of smell and taste. However, the subjective olfactory quality of life and ability to identify odorants are affected.

## Limitation of study

The study sample is limited due to the COVID-19 pandemic and needs a bigger sample size in future research.

## Suggestion for future research

Follow-up should be advocated in the long term such as six months and one year to follow up on hyposmia/anosmia patients. The Malay version of s QOD-NS questionnaire should be administered in all sinonasal-related surgery.

## Data Availability

The datasets used and/or analyzed during the current study are available from the corresponding author on reasonable request.
